# Heavy Metal Susceptibility of Escherichia coli Isolated from Urine Samples from Sweden, Germany, and Spain

**DOI:** 10.1128/AAC.00209-18

**Published:** 2018-04-26

**Authors:** Susanne Sütterlin, Carlos J. Téllez-Castillo, Leticia Anselem, Hong Yin, James E. Bray, Martin C. J. Maiden

**Affiliations:** a Department of Zoology, University of Oxford, Oxford, United Kingdom; b Department of Clinical Microbiology, MVZ synlab Leverkusen GmbH, Köln-Merheim, Germany; c Department of Clinical Microbiology, Hospital Francesc De Borja De Gandía, Gandía, Spain; d Department of Clinical Microbiology, Falu Hospital, Falun, Sweden

**Keywords:** arsenic, heavy metal resistance, antibiotic resistance, *Escherichia coli*, silver

## Abstract

Antimicrobial resistance is a major health care problem, with the intensive use of heavy metals and biocides recently identified as a potential factor contributing to the aggravation of this situation. The present study investigated heavy metal susceptibility and genetic resistance determinants in Escherichia coli isolated from clinical urine samples from Sweden, Germany, and Spain. A total of 186 isolates were tested for their sodium arsenite, silver nitrate, and copper(II) sulfate MICs. In addition, 88 of these isolates were subjected to whole-genome sequencing for characterization of their genetic resistance determinants and epidemiology. For sodium arsenite, the isolates could be categorized into a resistant and a nonresistant group based on MIC values. Isolates of the resistant group exhibited the chromosomal *ars* operon and belonged to non-B2 phylogenetic groups; in contrast, within the B2 phylogroup, no *ars* operon was found, and the isolates were susceptible to sodium arsenite. Two isolates also harbored the silver/copper resistance determinant *pco/sil*, and they belonged to sequence types ST10 (phylogroup A) and ST295 (phylogroup C). The ST295 isolate had a silver nitrate MIC of ≥512 mg/liter and additionally produced extended-spectrum beta-lactamases. To our knowledge, this is the first study to describe the distribution of the arsenic resistance *ars* operon within phylogroups of E. coli strains isolated from patients with urinary tract infections. The arsenic resistance *ars* operon was present only in all non-B2 clades, which have previously been associated with the environment and commensalism in both humans and animals, while B2 clades lacked the *ars* operon.

## INTRODUCTION

Infections with multiresistant bacteria have rapidly increased in both hospitals and the community during the first decades of the 21st century. Extended-spectrum beta-lactamase (ESBL)-producing members of the Enterobacteriaceae family are part of this development. They account for two-thirds of the in-hospital mortality in Europe, and this proportion is increasing (1). One of the most important members of this family is Escherichia coli, a common colonizer of the intestinal tracts of humans and animals and the leading cause of urinary tract infections (UTIs) and Gram-negative sepsis (2). An association between the use of heavy metals and the development of antibiotic resistance has been recognized since at least the 1970s (3, 4). Heavy metals are used as antiseptics, disinfectants, and preservatives in health care and consumer products (5, 6), are also used in animal food production (7, 8), and accumulate as part of industrial waste in soils (9, 10). Thus, these substances contribute significantly to environmental pollution, with potential harm to wildlife and, consequently, humans (9–11). The human microbiota is increasingly exposed to these chemicals through a wide range of products, drinking water, and the food chain (5, 11).

Genetic determinants leading to resistance to arsenic, copper, or silver are widely distributed in environmental bacteria but also in human pathogens (6, 12). Likewise, beta-lactamases are widespread within many bacterial taxa, and due to increased antimicrobial selection pressure, some of these chromosomal genetic determinants have become associated with mobile genetic elements. For example, the chromosomal extended-spectrum beta-lactamase CTX-M gene from Kluyvera sp. has been mobilized at least once and successfully spread to E. coli and Klebsiella pneumoniae, causing the ongoing CTX-M pandemic (13, 14). Similar to the situation for antimicrobial resistance, there are reports of heavy metal-resistant bacteria related to the (mis)use of these substances in animal husbandry (8, 15) and hospital environments (16–18).

A great concern is the potential coselection of resistance to antimicrobials caused by exposure to heavy metals. There are reports of isolates with combined resistance to both antibiotics and heavy metals (6). In the human gut, heavy metal exposure levels are sublethal for bacteria, making them possible resistance-driving mechanisms (19). Nonspecific mechanisms, such as reduced permeation ability or uptake and enhanced efflux (20, 21) or shared mobile genetic elements (15, 22–24), are able to mediate resistance to both heavy metals and antimicrobials. However, although such coselection seems likely (15, 22, 23, 25), this is poorly understood. Worryingly, clinical isolates with combined heavy metal and antimicrobial resistance have regularly been found to be involved in hospital outbreak situations (18, 23, 26). It is hard to predict what impact increased exposure to heavy metals has on the development and spread of new potent clinical isolates, especially because resistance data for heavy metals are only infrequently obtained.

Here we investigate the resistances to several heavy metals and antibiotics among E. coli isolates from urine samples collected from patients at three hospitals situated in different parts of Europe. Using whole-genome sequence (WGS) analysis, parts of the isolate collection were further characterized regarding genetic antimicrobial resistance determinants and clinically important epidemiological markers.

## RESULTS

### Susceptibility testing. (i) Antimicrobials.

Resistance to the tested antibiotics was rare, with the exception of ampicillin, trimethoprim, co-trimoxazole, and ciprofloxacin. For these antibiotics, the lowest rates of susceptible isolates were found in Spanish isolates. A total of 19 isolates (9.9%) produced ESBL-type enzymes. Nine isolates were from Spain, three from Germany, and seven from Sweden. Eleven of these isolates were included in the whole-genome sequence analysis. Of these, all isolates carried ESBLs of the CTX-M type, including CTX-M-1 (*n* = 1), CTX-M-9 or CTX-M-9-like (*n* = 2), CTX-M-15 (*n* = 5), CTX-M-27 (*n* = 1), CTX-M-55 (*n* = 1), and both CTX-M-15 and CTX-M-27 (*n* = 1). Additionally, OXA-1 enzymes were found in three isolates with CTX-M production and one isolate without an ESBL phenotype (see Table S2 in the supplemental material).

### (ii) MIC distributions for heavy metals.

The heavy metal MIC ranges for the 186 isolates analyzed were as follows: for sodium arsenite, 8 to 2,048 mg/liter; for silver nitrate, 8 to >512 mg/liter; and for copper(II) sulfate, 512 to 2,048 mg/liter (Table 1). For sodium arsenite, the study isolates could be categorized into two groups, with a cutoff at 128 mg/liter: 67 isolates had MICs of >128 mg/liter and 119 isolates had MICs of ≤128 mg/liter. For silver nitrate, isolate WTCHG-295 had a MIC of >512 mg/liter when retested by macrodilution assay (Fig. S1.a to c).

**TABLE 1:** Results of MIC testing for biocides and heavy metals on study isolates and three reference strains

Compound	MIC result (mg/liter) for study isolates (*n* = 186 E. coli isolates)			MIC ranges (mg/liter) for reference strains^*a*^		
	Range	MIC_50_	MIC_90_	E. coli ATCC 25922	E. cloacae CCUG 38136	K. pneumoniae ATCC 700603
Sodium arsenite	8–−2,048	64	1,024	32–128	32–128	1,024
Silver nitrate	8–>512	32	32	16–32	16–32	16–32
Copper(II) sulfate	512–2,048	1,024	1,024	1,024–2,048	1,024–2,048	1,024–2,048

a The presented MIC ranges for all reference strains are based on microdilution assays only.

### Summary of the sequenced isolate collection. (i) Genome assembly.

Whole-genome sequencing was performed on a subcollection of 96 isolates. High-quality short reads for E. coli were obtained for 88 isolates, with an average coverage of 108 (standard deviation [SD] of ±23). Draft genomes were obtained for all sequenced isolates, using kmer values of 99 to 129 (median of 121), resulting in a median contig number of 162 (minimum and maximum values, 49 and 550) and a median *N*_50_ of 208,148 (minimum and maximum values, 59,334 and 681,908). The average genome length was 5,139,676 ± 198,236.

### (ii) Phylogenetic groups and sequence types.

Among the 88 sequenced isolates, 55 isolates (62.5%) were assigned to phylogroup B2, 13 isolates (15%) to phylogroup D, and 18 isolates (20%) to phylogroup B1, F, C, E, or A. The most frequent sequence types were ST131 (phylogroup B2) (*n* = 15), ST73 (B2) (*n* = 13), ST69 (D) (*n* = 6), ST95 (B2) (*n* = 5), ST1193 (B2) (*n* = 5), ST127 (B2) (*n* = 4), and ST141 (B2) (*n* = 3). Among the CTX-M-producing isolates, the most frequent sequence type was ST131 (B2; CTX-M-15 [*n* = 4], CTX-M-27 [*n* = 1], and CTX-M-15 and -27 [*n* = 1]), and for the remaining ESBL-producing isolates, the sequence types were ST38 (D; CTX-M-9), ST156 (B1; CTX-M-1), ST115 (D; CTX-M-9), ST23 (C; CTX-M-55), and ST295 (C; CTX-M-15). The sequence types were equally distributed among countries, patient genders, and patient ages. No statistically significant associations of antimicrobial susceptibility and heavy metals were observed, but both *pco/sil* and *ars* resistance determinants showed an association with non-B2 phylogroups.

### Genetic and phenotypic resistance to heavy metals.

**FIG 1:**
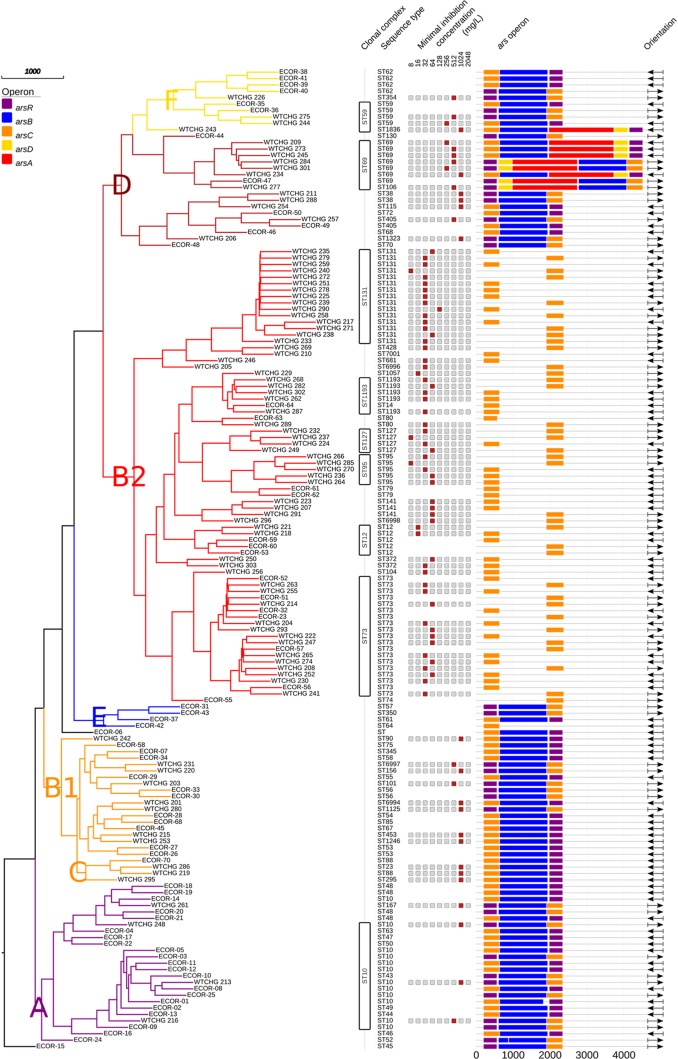
Neighbor-joining tree based on 51 rMLST allele sequences for study isolates and the ECOR strain collection. Colored leaves in the tree indicate phylogenetic groups (F, D, B2, E, B1, C, and A). Columns show the following, from left to right: clonal complexes and sequence types according to seven-gene MLST (Achtman scheme), MICs for sodium arsenic (a red box indicates the MIC for the respective isolate, gray boxes are placeholders, and blank lines mean that no data were available), *ars* operons (purple, *arsR*; blue, *arsB*; orange, *arsC*; yellow, *arsD*; and red, *arsA*), and orientations in the draft genome (→, forward; and ←, reverse).

All isolates with sodium arsenite MICs of >128 mg/liter exhibited the chromosomal *ars* operon *arsRBC* or *arsRDABC* (*n* = 36). In contrast, all study isolates that lacked the *arsRB* determinants had MICs of <128 mg/liter. None of the isolates investigated harbored plasmid-borne *ars* operons. The complete *arsRDABC* operon was found in isolates belonging to the ST69 complex (*n* = 7) and in one isolate assigned to ST1836 (WTCHG_243); both these sequence types belong to phylogroup D. The *arsRBC* determinants were found in all other non-B2 groups. These findings were confirmed with the isolates within the ECOR strain collection, with the exception that no *ars* operon was found in ECOR-42 (ST64) (Fig. 1).

Two isolates were positive for the silver resistance determinant *sil/pco*. One (WTCHG-248) belonged to ST10 (phylogroup A) and the other (WTCHG-295) to ST295 (phylogroup C); the latter also harbored CTX-M-15 enzymes and was positive for the quinolone efflux pump gene *qnrS*. The MIC of silver nitrate in microdilution assays was 16 mg/liter for both isolates, but isolate WTCHG-295 had a MIC of >512 mg/liter when retested by macrodilution assay. Isolate WTCHG-295 even grew after one passage in silver nitrate at >512 mg/liter.

### *ars* operon.

**FIG 2:**
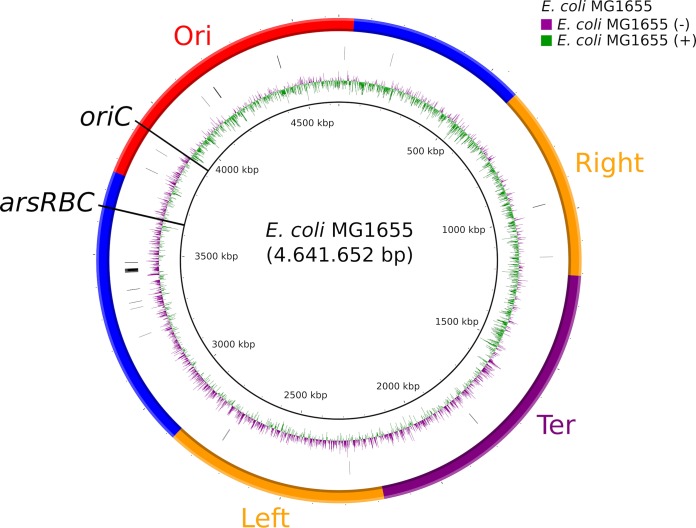
Location of the *ars* operon on the chromosome of E. coli MG1655. The image was constructed using BRIG. The inner to outer circles indicate the scale (kilobase pairs), GC skew of E. coli MG1655, positions of ribosomal genes, and the macrodomains Ori, Right, Ter, and Left as well as less structured regions (as described by Valens et al. [58]).

**FIG 3:**
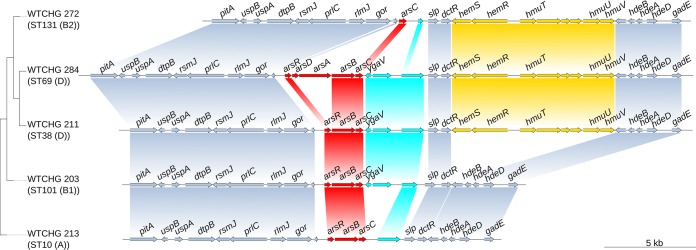
Gene synteny of *ars* operons and their genetic environment for the following five representative isolates (phylogroup): WTCHG_213 (A), WTCHG_203 (B1), WTCHG_211 (D), WTCHG_284 (D), and WTCHG_272 (B2).

**FIG 4:**
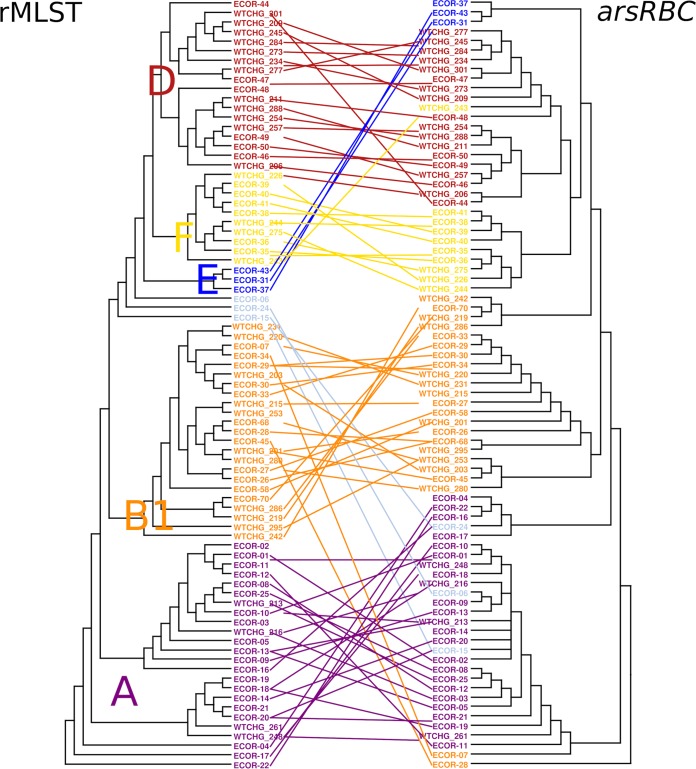
Tangle plot of neighbor-joining trees for rMLST and *arsRBC* sequences. The rMLST tree is based on 51 rMLST allele sequences (left) and concatenated *arsRBC* sequences (right) for *ars*-positive isolates from the study isolate collection and the ECOR strain collection. Colors and letters indicate phylogenetic groups (D, F, E, B1, and A).

A closer examination of the genetic context of the *ars* operon showed that in E. coli MG1655, the *ars* determinants were located around 275 kbp from the replication origin (Fig. 2). The gene content of the operon and the surrounding genes showed associations with phylogenetic groups, as shown in Fig. 3 for one representative isolate per phylogroup. In B2 phylogroup isolates, the region between *gor* and the following open reading frame (ORF), up until *slp*, contained only the arsenic reductase gene, *arsC*. Despite the fact that the *ars* operons of the isolates belonging to phylogroup B2, ST69 (phylogroup D), and all other sequence types of phylogroups D and F were different, all of these isolates possessed the *hemSR* and *hmuTUV* genetic determinants, genes that are thought to be involved in iron transport, around 5 kbp downstream of the *ars* operon (Table S3). Within this study, phylogroups E and C were underrepresented, but the composition of the genetic environment of the *ars* operon for these phylogroups strongly resembled that for the neighboring groups of the ribosomal multilocus sequence typing (rMLST) tree. Phylogroup E isolates possessed the gene order of either B1 or B2 isolates, and phylogroup C isolates possessed the gene order of either phylogroup A or B1 isolates (Fig. 3). The phylogenetic categorization based on concatenated ribosomal sequences and that derived from the *arsRBC* genes resulted in identical phylogroup assignments for most isolates (Fig. 4).

## DISCUSSION

In the present study, heavy metal resistance in E. coli isolates from urine samples was strongly associated with phylogenetic clades which have previously been associated with the environment and commensalism and thus reflect the selective pressure to which bacteria are exposed. A particularly strong association was found for arsenic, and isolates could be categorized into two subgroups according to their arsenic susceptibility. The isolates in the more resistant group possessed the chromosomal *ars* operon and belonged to non-B2 phylogenetic groups of E. coli, which have previously been associated with the environment and commensalism, whereas the arsenic-susceptible group did not. Although it was less frequent, resistance to silver mediated by the copper/silver resistance determinant *pco/sil* in E. coli also occurred only in non-B2 phylogroups.

Arsenic is a heavy metal that is ubiquitous in the environment, where bacteria are exposed to it at various levels. Resistance to arsenic is therefore common in a wide range of bacteria and can be found in many species within the Enterobacteriaceae family (6). Within the study isolates, 36% had an arsenic resistance phenotype, with a suggested epidemiological cutoff (ECOFF) value of 128 mg/liter. This phenotypic arsenic resistance was associated with the occurrence of the chromosomal *ars* operon. Interestingly, although arsenic resistance genes were previously described mainly for plasmids (27–29), there were no plasmid-borne arsenic resistance genes in these isolates. Furthermore, isolates with increased tolerance to arsenic all belonged to non-B2 phylogroups. Because of environmental pollution, humans and the gut microbiota are exposed to increasing levels of arsenic through drinking water and the food chain (9, 11); however, while the enzymes mediated by the bacterial *ars* operons reduce the toxicity of their ligands, As(III) and As(V), human exposure is due mainly to arsenic bound in organic compounds (11). Furthermore, clearance of absorbed arsenic from the human body occurs mainly via the renal route, with only minimal hepatobiliary excretion (11). Therefore, it is likely that the gut microbiota is exposed to comparatively low arsenic levels, which additionally are bound in compounds that are not ligands of the enzymes encoded by the *ars* operon.

E. coli strains belonging to phylogroup B2 are known to be associated with extraintestinal infections and thus are well adapted to the human host (2). Hence, it is consistent that the lack of tolerance to arsenic in E. coli isolates belonging to the B2 phylogroup does not lead to a survival disadvantage, and thus the deletion of *arsR* and *arsB* might have been beneficial to this group. In contrast, our findings suggest that E. coli strains belonging to commensal and environmental phylogroups, with a higher risk of exposure to As(III) and As(IV) redox states, may well have a survival advantage from arsenic resistance factors encoded by the *ars* operon (9). Interestingly, there was a small group of isolates within phylogroup D that carried *arsD* and *arsA* in addition to *arsRBC*. Both *arsD* and *arsA* are thought to give competitive benefits in environments with high arsenic levels (30), as well as increased fitness compared to that of isolates with only *arsRBC* genes (31); however, among our study isolates, there was no significant difference regarding the sodium arsenite MIC between isolates with and without *arsD* and *arsA*.

All isolates possessed the arsenate reductase encoded by *arsC*, which catalyzes the reduction of arsenate to the less toxic compound arsenite (9). The genetic environment of *arsC* in combination with the finding that the phylogenies of the concatenated sequences of ribosomal genes and *arsRBC* were the same supported the conclusion that *arsR* and *arsB* were lost in phylogroup B2 isolates rather than acquired in non-B2 isolates. While the deletion of the arsenite transporter gene *arsB* and the transcriptional repressor gene *arsR* seemed to be a beneficial event for phylogroup B2 E. coli isolates, the *arsC* gene remained in all clades. Interestingly, the location of the *ars* operon on the E. coli chromosome is in close proximity to the origin of replication. The position of genes on the chromosome has previously been shown to influence gene expression (32), and proximity to the replication origin potentially increases expression (33). Thus, the proximity to the replication origin may indicate a particular biological relevance of *ars* genes to bacteria. Although *arsC* was found in all isolates, it remains unclear whether that can be explained by the ubiquitous occurrence of arsenic in the environment or whether *arsC* has an additional, unknown biological function.

While arsenic is one of the most ubiquitous of all environmental toxic compounds, silver is rapidly bound to proteins or halogens or reduced to atomic silver and thus is less abundant in its toxic form than arsenic. This is reflected in the dissemination of systems of resistance to silver ions: the plasmid-borne silver resistance determinant *sil/pco* is regularly found in areas where silver compounds are used or released (34–36) and less frequently observed in E. coli populations from remote areas (22). This is of particular interest because silver compounds are still used as antimicrobials in hospitals and patient care. The interest in copper as an antimicrobial has also been renewed against the background of increasing antibiotic resistance. In contrast to arsenic and silver, copper is essential for cell physiology, and thus sophisticated systems to maintain homeostasis are present in bacterial cells (37). The plasmid-borne *pco* operon was previously shown to increase tolerance to copper ions (38) but not to copper alloys, which are becoming increasingly popular as antimicrobial surface materials in hospital environments (39). Copper-resistant bacteria have been described as an emerging problem within animal husbandry, where copper is widely used as a food additive (40). However, while silver-resistant isolates are frequently isolated from patients, to our knowledge, this has not been described for copper resistance.

Arsenic, silver, and copper are potential resistance-driving factors in *in vitro* studies (41, 42). This is also clinically relevant, since isolates with *sil/pco* determinants regularly coproduce ESBL enzymes (15, 22, 23). The studies mentioned above specifically highlighted the role of horizontal gene transfer via plasmids, and thus coselection for spread of resistance determinants. Similar to previous findings, one of the *sil/pco*-positive isolates from this study also produced CTX-M-15 enzymes. The present study emphasizes the role of clones in spread of resistance: phylogroup B2 was previously found to be less resistant to antimicrobials (43), which is in line with our findings regarding arsenic susceptibility. Worryingly, environmental pollution with arsenic has a significant influence on the gut microbiota, leading to a shift toward increased resistance (44). One can only speculate about the contribution of this selective pressure to the risk of development of new virulent and multiresistant clones.

In conclusion, the distribution of arsenic and silver resistance phenotypes in E. coli strains is concentrated among E. coli phylogroups associated with the environment rather than among specific pathotypes. Thus, the substantial environmental pollution with arsenic, silver, and antimicrobials in some areas of the world may pose a risk for the development of isolates carrying multiple resistance genes. The phylogenetic relationships described in the present study may contribute to a better understanding of the resistance potential and selection mechanisms of pathogenic E. coli in human and veterinary settings.

## MATERIALS AND METHODS

### Bacterial isolates.

A total of 186 E. coli isolates from urine samples were included in the study. These were collected during spring and early summer 2016 from three hospitals, in Falun, Sweden (May-June 2016); Bautzen, Germany (March-April 2016); and Gandía, Spain (April to July 2016). Each participating clinic collected E. coli isolates from female and male patients successively until isolates had been recovered from 100 females. Since UTIs are more common in females than in males, more isolates were collected from females than from males during the collection periods. To avoid an imbalance by gender, isolates from females were randomly chosen to match the number of isolates from males, as follows: 30 isolates from each gender in Falun, 29 isolates from each gender in Bautzen, and 34 isolates from each gender in Gandía. Each patient's age, gender, and hospital ward were recorded, and the isolates were anonymized; therefore, no consent from the patients was required.

Standard laboratory procedures and automated species identification systems were used to identify the bacteria to the species level (BD Phoenix automated microbiology system [BD, USA] in Gandía, MicroScan Walkway system [Beckman Coulter, USA] in Bautzen, and matrix-assisted laser desorption ionization–time of flight [MALDI-TOF] analysis [Bruker Daltonics, USA] in Falun). Isolates were frozen as glycerol stocks at −80°C prior to further analysis.

### Susceptibilities to heavy metals and antibiotics. (i) Antibiotic susceptibility testing.

Antibiotic susceptibility testing was performed by the disc diffusion method as recommended by EUCAST (http://www.eucast.org). The following antibiotics were included: ampicillin, piperacillin-tazobactam, amdinocillin, cefadroxil, cephalexin, cefepime, cefotaxime, ceftazidime, ceftibuten, cefuroxime, ertapenem, meropenem, imipenem, aztreonam, ciprofloxacin, nalidixic acid, gentamicin, tobramycin, amikacin, tigecycline, nitrofurantoin, trimethoprim, trimethoprim-sulfamethoxazole, and chloramphenicol. The isolates were categorized as susceptible, intermediate, or resistant by using the species-related breakpoints defined by EUCAST. The first criterion for testing for production of ESBLs was resistance to cefadroxil (inhibition zone of <12 mm) or cephalexin (inhibition zone of <14 mm). To confirm ESBL production phenotypically, a disc diffusion synergy test with clavulanic acid and cefotaxime, ceftazidime, or cefepime was used (http://www.nordicast.org).

### (ii) Heavy metal susceptibility testing.

To test the susceptibility of isolates to heavy metals and biocides, the MICs were determined in a microdilution assay (100 μl) performed according to the ISO 20776-1:2006 method (45), with the exception that Iso-Sensitest broth (Oxoid, United Kingdom) was used. The following substances and concentration ranges were included: sodium arsenite, 4 to 2,048 mg/liter; silver nitrate, 4 to 128 mg/liter; and copper(II) sulfate, 128 to 4,096 mg/liter (all from Sigma-Aldrich, USA). All isolates with elevated MICs for silver nitrate (≥64 mg/liter) were retested in the macrodilution format (1 ml) in glass tubes. Stock solutions were prepared freshly at all times. Serial dilutions were inoculated with bacteria within 2 h after preparation. Following overnight culture in 1.5 ml Luria-Bertani broth (Sigma-Aldrich, USA) in the ambient atmosphere at 35°C, a bacterial suspension resulting in a final inoculum of 5 × 10^4^ CFU for microdilution or 5 × 10^5^ CFU for macrodilution was prepared. The plates and tubes were incubated for 18 to 20 h in the ambient atmosphere at 35°C, and the MICs were read as the lowest concentrations yielding no visible growth. E. coli ATCC 25922, Enterobacter cloacae CCUG 38138, and Klebsiella pneumoniae ATCC 700603 were used as control strains.

### Whole-genome analysis. (i) DNA preparation and whole-genome sequencing.

A randomly chosen subcollection of the isolates tested for susceptibility (*n* = 96) were analyzed by whole-genome sequencing (WGS). One colony of each isolate was incubated in 2 ml Luria-Bertani broth for 8 h at 37°C. DNA was prepared by use of a Wizard Genomic DNA purification kit (Promega, USA) according to the manufacturer's recommendations for Gram-negative bacteria, with the exception that DNA was rehydrated with 10 mM Tris-HCl (pH 8.0). The quality and quantity of the extracted DNA were assessed by gel electrophoresis, spectrophotometry (NanoDrop; Thermo Fisher), and a Quant-iT dsDNA BR assay with a Qubit instrument (Invitrogen, USA). After standardization of the DNA extracts, the samples were transferred to the Oxford Genome Center for library preparation and WGS. Briefly, fragmented DNAs were end repaired, A-tailed, adapter ligated, and amplified using a Nextera DNA library prep kit (Illumina, USA). Sequencing was done on an Illumina HiSeq4000 platform, generating 150-bp paired-end reads.

### (ii) Sequence analysis.

The read quality was assessed using FastQC software (v0.11.4; http://www.bioinformatics.babraham.ac.uk) according to the developers' recommendations. Species identification was performed using the rMLST tool available at http://pubmlst.org/rmlst. This tool searches for exact matches to sequences defined in the rMLST allele library, which are derived from more than 7,000 bacterial species. Allelic matches are cross-referenced with a large curated set of bacterial isolates to determine the most likely species present in the DNA sample. Illumina short reads were mapped to databases for resistance genes (ARG-ANNOT, V2 [December 2015]) (46) for antibiotic resistance genes and to a database for mainly plasmid-borne heavy metal resistance genes that have been described thoroughly (see Table S1 in the supplemental material), using srst2 (v0.2.0) (47).

The isolates were assigned to the main E. coli phylogenetic groups on the basis of their clustering in a neighbor-joining tree created from rMLST alleles. Sequences were assembled using Velvet (v1.2.10) and VelvetOptimiser (v2.2.4) software, with sampling of all odd kmer lengths from 21 to 149. The default optimization parameters were used, together with a minimum contig size of 200 bp and the scaffolding option switched off. Using BIGSdb database software (48), multilocus sequence typing was performed according to the seven-gene Achtman scheme (49). A neighbor-joining tree was constructed for rMLST (50) allele nucleotide sequences of the study isolates and the E. coli reference collection (ECOR; sequences were obtained from the ENA/SRA/DDBJ databases) (51, 52). Concatenated sequences for the rMLST scheme were retrieved and aligned by use of MAFFT (v7.271) (53), and the tree was calculated using PHYLIP (v3.695) (54). Paralogous loci were excluded (BACT000060 and BACT000065), resulting in 51 concatenated ribosomal loci for the rMLST analysis. The data set was then bootstrapped 500 times with PHYLIP SEQBOOT, followed by calculations of distance matrices with PHYLIP DNADIST and drawing of neighbor-joining trees with PHYLIP NEIGHBOR and of a consensus tree with PHYLIP CONSENSE.

Draft genomes were annotated by use of the annotation software Prokka (v1.12-beta) (55) with default settings, followed by a parsing of the output files for the *ars* operon (coding sequences) for each isolate, using the SeqIO module in Biopython (56) (parsing script available on request). For *ars*-positive isolates, a neighbor-joining tree was calculated based on concatenated sequences for *arsRBC* and rMLST in accordance with the procedure described for the rMLST tree described above. Both trees were compared after construction of a tanglegram by use of Dendroscope software (v3.5.9; http://dendroscope.org).

### Data handling and statistics.

The results from the susceptibility testing with antimicrobial substances and from the short-read mapping (resistance determinants) were analyzed by focusing on coresistance and the influence of gender, age, and origin. In addition, the frequencies of resistance determinants and phenotypes were evaluated with regard to phylogenetic groups and sequence types.

Associations between the variables were measured using Pearson correlation (phi coefficient for binary variables). For all variable pairs with correlation or phi coefficients of >0.5 or <−0.5, the correlation was checked for meaningfulness. For meaningful associations, hypotheses were formulated, the odds ratio (OR) was calculated, and statistical significance was evaluated by Fisher's exact test. Results with ORs of >2 or <0.5 and *P* values of <0.05 (statistical significance) were included.

Phylograms were visualized using EvolView (http://www.evolgenius.info), and histograms and gene synteny were visualized using the statistical software R (v3.3.3; packages ggplot2 and genoPlotR) (R Foundation for Statistical Computing, Vienna, Austria). The location of the *ars* operon on the E. coli chromosome was visualized using the BLAST Ring Image Generator (BRIG) (v0.95) (57), with the finished chromosome sequence of E. coli MG1655 (GenBank accession number U00096.3) as a reference.

### Accession number(s).

All 88 paired-end reads are available from the ENA/SRA/DDBJ databases under project reference PRJEB17631. See Table S4 in the supplemental material for individual accession numbers.

## Supplementary Material

Supplemental material
