# Supplementary material for: Heavy Metal Susceptibility of Escherichia coli Isolated from Urine Samples from Sweden, Germany, and Spain
Source: Antimicrob Agents Chemother. 2018 Apr 26;62(5):e00209-18. doi: 10.1128/AAC.00209-18 (PMC5923176; doi:10.1128/AAC.00209-18)
Supplement: Supplemental material [file supp_62_5_e00209-18__index.html]

Supplemental material 

# Heavy Metal Susceptibility of Escherichia coli Isolated from Urine Samples from Sweden, Germany, and Spain

## Supplemental material

- Supplemental file 1 -

  Tables S1 to S4 and Fig. S1

  PDF, 1.6M
