# Supplementary material for: Heavy Metal Susceptibility of Escherichia coli Isolated from Urine Samples from Sweden, Germany, and Spain
Source: Antimicrob Agents Chemother. 2018 Apr 26;62(5):e00209-18. doi: 10.1128/AAC.00209-18 (PMC5923176; doi:10.1128/AAC.00209-18)
Supplement: Supplemental material [file AAC.00209-18_zac005187166s1.pdf]

Table S1. Overview over the nucleic acid sequences included in the srst2-analysis.

**Genes**	**Function**	**Accession number**	**Source**	**Ref.**
arsA,B,C,R,D	Arsenic resistance system (plasmidal)	J02591, U38947, AJ288983	R773, R478, R46	1-3
arsB,C,R	Arsenic resistance sytem (chromosomal)	X80057	*E. coli* K-12	4
silE,A,B,C,S,R,P	Silver resistance system	AF067954, CP002474	pMG101 (*S. typhimurium*), pUUH239 (*K. pneumonia*)	5, 6
pcoA,B,C,D,R,S,E	Copper resistance system	X83541	pRJ1004 (*E. coli*)	7
merA,B,D	Mercury resistance system	M15049	pDU1358 (from *S. marcesc*ens)	8
czcN,I,C,B,A,D,R,S	Cobalt, zinc, and cadmium resistance system	X98451	*Ralstonia sp*.	9
nreA,B	Nickel resistance determinant	L31491	pTOM9 (*Alcaligenes xylosoxidans*)	10
cnrY,X,H,C,D,B,A,T tnpA	Cobalt-nickel-resistance system	AJ276513	pMOL28 (*Ralstonia sp.*)	11
ncrA,B,C,Y,X	Nickel/cobalt resistance determinant	AF322866	pNRS148 (*Hafnia alvei*)	12

References

[1] Chen CM, Misra TK, Silver S, Rosen BP. 1986. Nucleotide sequence of the structural genes for an anion pump. The plasmid-encoded arsenical resistance operon. *J Biol Chem* 261: 15030-8.

[2] Ryan D, Colleran E. 2002. Arsenical resistance in the IncHI2 plasmids. *Plasmid* 47: 234-40.

[3] Bruhn DF, Li J, Silver S, Roberto F, Rosen BP. 1996. The arsenical resistance operon of IncN plasmid R46. *FEMS Microbiol Lett* 139: 149-53.

[4] Diorio C, Cai J, Marmor J, Shinder R, DuBow MS. 1995. An *Escherichia coli* chromosomal *ars* operon homolog is functional in arsenic detoxification and is conserved in gram-negative bacteria. *J Bacteriol* 177: 2050-6.

[5] Gupta A, Matsui K, Lo J-F, Silver S. 1999. Molecular basis for resistance to silver cations in *Salmonella*. *Nat Med* 5: 183-8.

[6] Sandegren L, Linkevicius M, Lytsy B, Melhus Å, Andersson DI. 2012. Transfer of an *Escherichia coli* ST131 multiresistance cassette has created a *Klebsiella pneumoniae*-specific plasmid associated with a major nosocomial outbreak. *J Antimicrob Chemother* 67: 74-83.

[7] Brown NL, Barrett SR, Camakaris J, Lee BT, Rouch DA. 1995. Molecular genetics and transport analysis of the copper-resistance determinant (*pco*) from *Escherichia coli* plasmid pRJ1004. *Mol Microbiol* 17: 1153-66.

[8] Griffin HG, Foster TJ, Silver S, Misra TK. 1987. Cloning and DNA sequence of the mercuric- and organomercurial-resistance determinants of plasmid pDU1358. *Proc Natl Acad Sci U S A* 84: 3112-6.

[9] Nies DH, Nies A, Chu L, Silver S. 1989. Expression and nucleotide sequence of a plasmid-determined divalent cation efflux system from Alcaligenes eutrophus. *Proc Natl Acad Sci U S A* 86: 7351-5.

[10] Schmidt T, Schlegel HG. 1994. Combined nickel-cobalt-cadmium resistance encoded by the *ncc* locus of *Alcaligenes xylosoxidans* 31A. *J Bacteriol* 176: 7045-54.

[11] Grass G, Grosse C, Nies DH. 2000. Regulation of the cnr cobalt and nickel resistance determinant from *Ralstonia sp*. strain CH34. *J Bacteriol* 182: 1390-8.

[12] Park JE, Schlegel HG, Rhie HG, Lee HS. 2004. Nucleotide sequence and expression of the *ncr* nickel and cobalt resistance in *Hafnia alvei* 5-5. *Int Microbiol* 7: 27-34.

Figure S1.a-c.

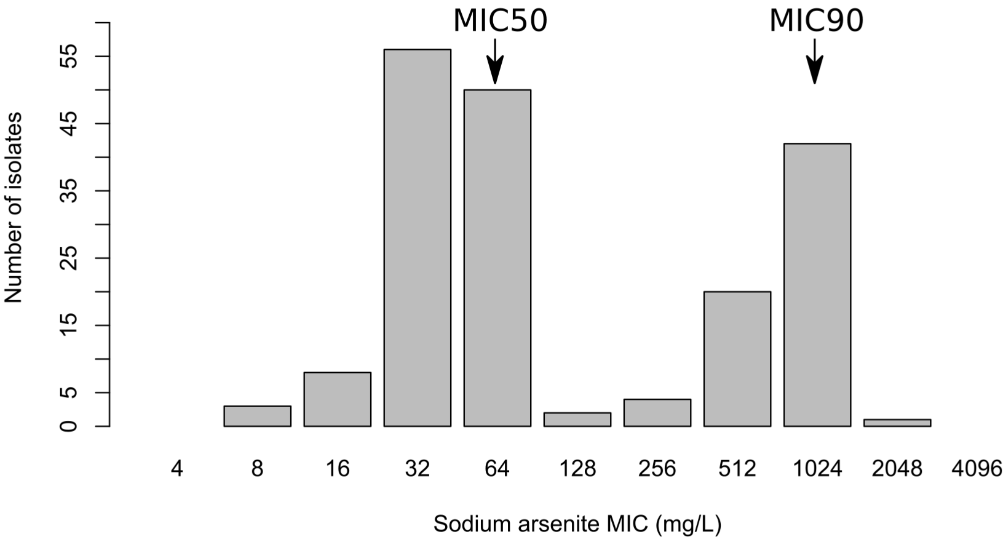

Figure 1.a

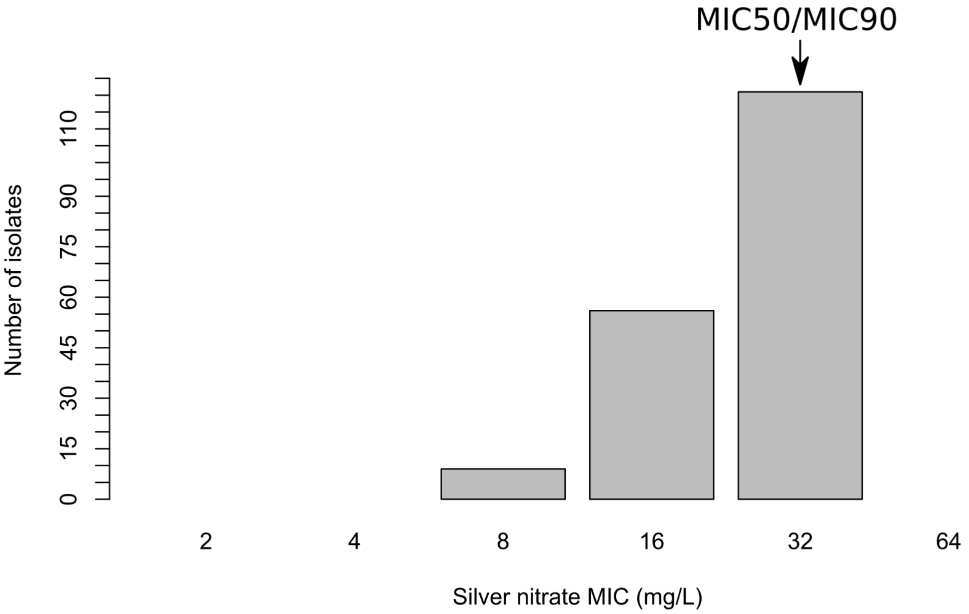

Figure 1b.

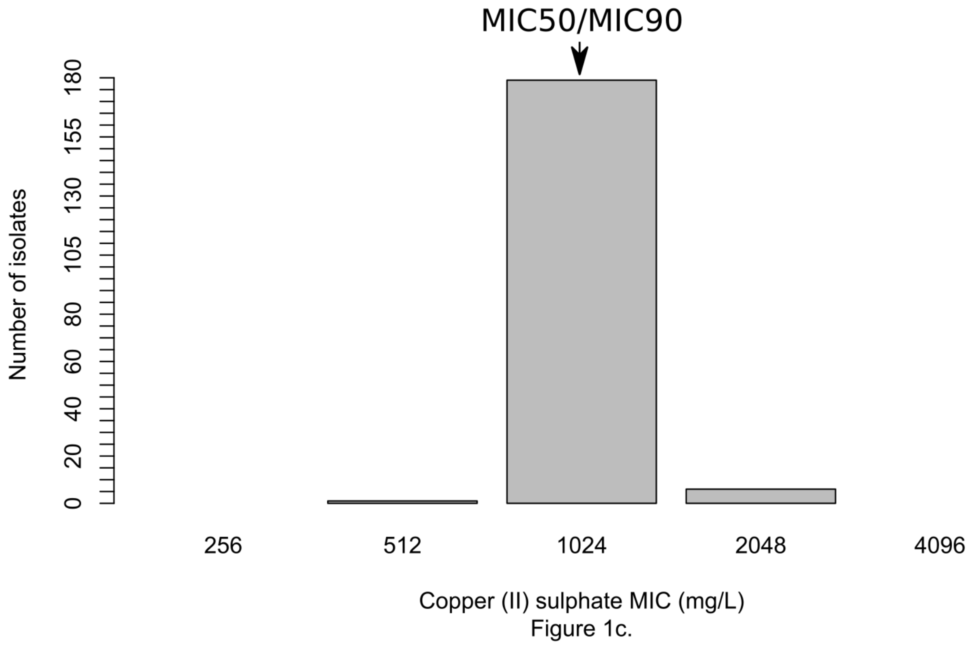

Figure S1. MIC distribution of the *E. coli* isolates included in the study for a) sodium arsenite b) silver nitrate, c) copper (II) sulphate

Table S2. Results on susceptibility testing for antimicrobials displayed for all study isolates and per origin.

	Total (n = 192)		Sweden (n = 60)		Germany (n = 58)		Spain (n = 74)	
	I/R[1] - absolute	S[2] -rate	I/R - absolute	S - rate	I/R - absolute	S -rate	I/R - absolute	S - rate
Ampicillin	96	50%	25	58%	24	59%	47	31%
Piperacillin-tazobactam	1	99%	1	98%	0	100%	0	100%
Mecillinam	4	98%	1	98%	1	98%	2	97%
Cefadroxil	21	89%	8	87%	3	95%	10	85%
Cefalexin	22	89%	9	85%	3	95%	10	85%
Cefepime	12	94%	7	88%	2	97%	3	96%
Cefotaxime	21	89%	8	87%	3	95%	10	85%
Ceftazidime	14	93%	8	87%	1	98%	5	95%
Ceftibuten	3	98%	1	98%	0	100%	2	97%
Cefuroxime	22	89%	8	87%	3	95%	11	84%
Ertapenem	0	100%	0	100%	0	100%	0	100%
Meropenem	0	100%	0	100%	0	100%	0	100%
Imipenem	0	100%	0	100%	0	100%	0	100%
Aztreonam	14	93%	8	87%	1	98%	5	93%
Ciprofloxacin	55	71%	11	82%	9	84%	35	49%
Nalidixid acid	78	59%	18	70%	16	72%	44	35%
Gentamicin	12	94%	1	98%	1	98%	10	85%

Tobramycin	14	93%	3	95%	1	98%	10	85%
Amikacin	0	100%	0	100%	0	100%	0	100%
Tigecycline	3	98%	2	97%	0	100%	1	99%
Nitrofurantoin	2	99%	2	97%	0	100%	0	100%
Trimethoprim	65	66%	15	75%	17	71%	33	51%
Co-trimoxazole	56	71%	13	78%	16	72%	27	60%
Chloramphenicol	11	94%	2	97%	5	91%	4	94%

Disc diffusion diameters translated according to EUCAST Breakpoint Tables v 6.0, [1] I/R – intermediate/resistant, [2] S – susceptible

Table S3. Genes and their putative proteins according to UniProt database (www.uniprot.org) surrounding the *ars* operon.

Gene[1]	Protein[2]	Funktion[2]	Evidence level[2]
pitA	Low-affinity inorganic phosphate transporter 1	Low-affinity inorganic phosphate transport. Can also transport arsenate	4
uspB	Universal stress protein B	Overexpression of *usp*B causes cell death in stationary phase.	3
uspA	Universal stress protein A	Required for resistance to DNA-damaging agents.	4
dtpB	Dipeptide and tripeptide permease B	Proton-dependent permease that transports di- and tripeptides.	2
rsmJ	Ribosomal RNA small subunit methyltransferase J	Specifically methylates the guanosine in position 1516 of 16S rRNA.	2
prlC	Oligopeptidase A		1
rlmJ	Ribosomal RNA large subunit methyltransferase J	Specifically methylates the adenine in position 2030 of 23S rRNA.	3
gor	Glutathione reductase		2
ygaV	Putative transcriptional regulator, ArsR family		1
slp	Outer membrane protein Slp		1
dctR	HTH-type transcriptional regulator DctR	May act as a transcriptional regulator of *dct*A	2
hemS	Hemin transport protein HmuS	Iron transport	1
hemR	TonB-dependent heme/hemoglobin receptor family protein<br>Outer membrane hemin receptor ChuA		1
hmuT	Putative periplasmic binding protein ChuT		1
hmuU	Iron chelate uptake ABC transporter, FeCT family, permease protein		1
hmuV	Hemin import ATP-binding protein HmuV	Part of the ABC transporter complex HmuTUV involved in hemin import. Responsible for energy coupling to the transport system.	2

hdeB	Acid stress chaperone HdeB	Required for optimal acid stress protection, which is important for survival of enteric bacteria in the acidic environment of the host stomach. Exhibits a chaperone-like activity at acidic pH by preventing the aggregation of many different periplasmic proteins.	2
hdeA	Acid stress chaperone HdeA	Required for optimal acid stress protection. Exhibits a chaperone-like activity only at low pH by suppressing non-specifically the aggregation of denaturated periplasmic proteins.	2
hdeD	Acid-resistance membrane protein, HdeD		1
gadE	Transcriptional regulator GadE	Regulates the expression of several genes involved in acid resistance. Required for the expression of *gad*A and *gad*BC, among others, regardless of media or growth conditions. Binds directly to the 20 bp GAD box found in the control regions of both loci.	3

[1] Gene as predicted by Prokka

[2] Description and evidence level according to Uniprot (www.uniprot.org)

Table S4. Data Availability: All 88 paired end reads are available from the ENA/SRA/DDBJ databases: Accession numbers: (ENA-number: study ID).

ERR1718808: WTCHG_201	ERR1718874: WTCHG_235	ERR1718932: WTCHG_265
ERR1718812: WTCHG_203	ERR1718876: WTCHG_236	ERR1718934: WTCHG_266
ERR1718814: WTCHG_204	ERR1718878: WTCHG_237	ERR1718938: WTCHG_268
ERR1718816: WTCHG_205	ERR1718880: WTCHG_238	ERR1718940: WTCHG_269
ERR1718818: WTCHG_206	ERR1718882: WTCHG_239	ERR1718942: WTCHG_270
ERR1718820: WTCHG_207	ERR1718884: WTCHG_240	ERR1718944: WTCHG_271
ERR1718822: WTCHG_208	ERR1718886: WTCHG_241	ERR1718946: WTCHG_272
ERR1718824: WTCHG_209	ERR1718888: WTCHG_242	ERR1718948: WTCHG_273
ERR1718826: WTCHG_210	ERR1718890: WTCHG_243	ERR1718950: WTCHG_274
ERR1718828: WTCHG_211	ERR1718892: WTCHG_244	ERR1718952: WTCHG_275
ERR1718832: WTCHG_213	ERR1718894: WTCHG_245	ERR1718956: WTCHG_277
ERR1718834: WTCHG_214	ERR1718896: WTCHG_246	ERR1718958: WTCHG_278
ERR1718836: WTCHG_215	ERR1718898: WTCHG_247	ERR1718960: WTCHG_279
ERR1718838: WTCHG_216	ERR1718900: WTCHG_248	ERR1718962: WTCHG_280
ERR1718840: WTCHG_217	ERR1718902: WTCHG_249	ERR1718966: WTCHG_282
ERR1718842: WTCHG_218	ERR1718904: WTCHG_250	ERR1718968: WTCHG_284
ERR1718844: WTCHG_219	ERR1718906: WTCHG_251	ERR1718970: WTCHG_285
ERR1718846: WTCHG_220	ERR1718908: WTCHG_252	ERR1718972: WTCHG_286
ERR1718848: WTCHG_221	ERR1718910: WTCHG_253	ERR1718974: WTCHG_287
ERR1718850: WTCHG_222	ERR1718912: WTCHG_254	ERR1718976: WTCHG_288
ERR1718852: WTCHG_223	ERR1718914: WTCHG_255	ERR1718978: WTCHG_289
ERR1718854: WTCHG_224	ERR1718916: WTCHG_256	ERR1718980: WTCHG_290
ERR1718856: WTCHG_225	ERR1718918: WTCHG_257	ERR1718982: WTCHG_291
ERR1718858: WTCHG_226	ERR1718920: WTCHG_258	ERR1718986: WTCHG_293
ERR1718862: WTCHG_229	ERR1718922: WTCHG_259	ERR1718990: WTCHG_295
ERR1718864: WTCHG_230	ERR1718924: WTCHG_261	ERR1718992: WTCHG_296
ERR1718866: WTCHG_231	ERR1718926: WTCHG_262	ERR1718994: WTCHG_301
ERR1718868: WTCHG_232	ERR1718928: WTCHG_263	ERR1718996: WTCHG_302
ERR1718870: WTCHG_233	ERR1718930: WTCHG_264	ERR1718998: WTCHG_303
ERR1718872: WTCHG_234		
